# Cytotoxic and Antimalarial Amaryllidaceae Alkaloids from the Bulbs of *Lycoris radiata*

**DOI:** 10.3390/molecules18032458

**Published:** 2013-02-25

**Authors:** Bin Hao, Shu-Fang Shen, Qing-Jie Zhao

**Affiliations:** 1 Department of Neurosurgery, Changhai Hospital, Second Military Medical University, Shanghai 200433, China; E-Mail: bhaosmmu@163.com; 2 Shanxi Children’s Hospital, Taiyuan 030012, Shanxi, China; 3 Department of Organic Chemistry, School of Pharmacy, Second Military Medical University, Shanghai 200433, China

**Keywords:** *Lycoris radiata*, Amaryllidaceae, alkaloids, cytotoxic, antimalarial

## Abstract

Phytochemical investigation of the 80% ethanol extract of the bulbs of *Lycoris radiata* resulted in the isolation of five new Amaryllidaceae alkaloids: (+)-5,6-dehydrolycorine (**1**), (+)-3*α,6β*-diacetyl-bulbispermine (**2**), (+)-3*α*-hydroxy-*6β*-acetyl-bulbispermine (**3**), (+)-8,9-methylenedioxylhomolycorine-*N*-oxide (**5**), and 5,6-dihydro-5-methyl-2-hydroxyphenanthridine (**7**), together with two known compounds, (+)-3*α*-methoxy-*6β*-acetylbulbispermine (**4**) and (+)-homolycorine- *N*-oxide (**6**). Structural elucidation of all the compounds were performed by spectral methods such as 1D and 2D (^1^H-^1^H COSY, HMQC, and HMBC) NMR spectroscopy, in addition to high resolution mass spectrometry. Alkaloid **1** showed potent cytotoxicity against astrocytoma and glioma cell lines (CCF-STTG1, CHG-5, SHG-44, and U251), as well as HL-60, SMMC-7721, and W480 cell lines with IC_50_ values of 9.4–11.6 μM. Additonally, compound **1** exhibited antimalarial activity with IC_50_ values of 2.3 μM for D-6 strain and 1.9 μM for W-2 strain of *Plasmodium falciparum*.

## 1. Introduction

**Figure 1:**
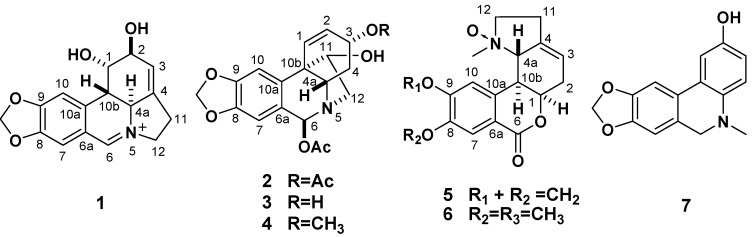
The structures of compounds **1**–**7**.

The genus *Lycoris* (Amaryllidaceae) consists of more than 20 species which are mainly distributed in the temperate woodlands of eastern Asia, particularly in China and Japan [1,2]. The alkaloids, the major chemical constituents of this plant genus, are known to have various chemical structures and a wide range of biological activities [3,4,5,6,7]. Alkaloids affect the central nervous system and have acetylcholinesterase-inhibitory, analgesic, anti-inflammatory, antiviral, antimalarial, antitumor, or antineoplastic activity [8,9,10,11,12,13,14]. Galantamine hydrobromide, derived from galanthamine, which is found in numerous Amaryllidaceae, has been clinically used for the treatment of Alzheimer’s disease [15]. *Lycoris* *radiata*, a perennial monocot, is endemic in China, Japan and Korea [16]. It is commonly known as Shi Shuan and used in China as a traditional folk medicine, from which more than ten indole alkaloids have been isolated [17,18]. The previous phytochemical studies revealed that *L. radiata* contained crinine-, galanthamine-, lycorine-, homolycorine- and montanine-type alkaloids [19,20]. The present studies on chemical constituents of the EtOH extract of *L. radiata* afforded five new Amaryllidaceae alkaloids, (+)-5,6-dehydrolycorine (**1**), (+)-3*α,*6*β*-diacetyl-bulbispermine (**2**), (+)-3*α*-hydroxy- 6*β*-acetylbulbispermine (**3**), (+)-8,9-methylenedioxyl-homolycorine-*N*-oxide (**5**), and 5,6-dihydro-5- methyl-2-hydroxyphenanthridine (**7**), and two known compounds, (+)-3*α*-methoxy-6*β*-acetyl-bulbispermine (**4**) and (+)-homolycorine-*N*-oxide (**6**) (Figure 1). In this paper, we describe the isolation and structure elucidation on the basis of spectroscopic methods of the new compounds. Furthermore, all the alkaloids were evaluated *in vitro* for their cytotoxic and antimalarial properties.

## 2. Results and Discussion

**Figure 2:**
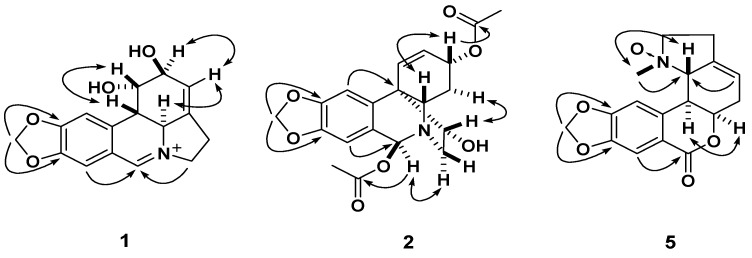
The key HMBC (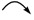) and ^1^H-^1^H COSY (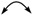) correlations of compounds **1**, **2**, and **5**.

Compound **1** was obtained as a yellow amorphous powder. The ESIMS afforded a quasimolecular ion peak at *m/z* 286, and its HR-ESI-MS revealed the [*M*]^+^ peak at *m/z* 286.1075 (calcd. for C_16_H_16_NO_4_^+^. 286.1074), corresponding to the molecular formula C_16_H_16_NO_4_^+^. Its UV absorption at λ_max_ 374, 309, 253, and 212 nm showed an extended chromophore and a methylenedioxyl substituted benzene ring. The IR absorption bands at 3,410, 3,355, 1,645, 1,605 and 923 cm^−1^ indicated OH groups and phenyl functions. The ^1^H-NMR spectrum of **1** exhibited two singlets for two *para*-located aromatic protons at *δ*_H_ 7.28 (H-7) and 7.18 (H-10), a methylenedioxy signal at *δ*_H_ 6.17 and a downfield singlet corresponding to the proton of an iminium salt (*δ*_H_ 8.84) [21]. The ^13^C-NMR spectrum showed 16 carbon signals [OCH_2_O × 1, CH_2_ (sp^3^) × 2, CH (sp^3^) × 4, CH (sp^2^) × 4 and C (sp^2^) × 5, Table 1]. The above data suggested that **1** was an amaryllidaceae alkaloid similar to lycorine [22], except for an imine moiety located between *N*-5 and C-6 (*δ*_C_ 163.2) in **1**, as supported by HMBCs of *δ*_H_ 8.84 (H-6) with *δ*_C_ 60.4 (C-4a), 113.0 (C-7), 128.3 (C-10a) and 59.0 (C-12) (Figure 2). The relative configuration of H-4a and H-10b in the amaryllidaceae alkaloids isolated from the genus *Lycoris* were always *α*- and *β*-orientations, respectively [22]. The relative configuration of **1** was elucidated by a ROESY experiment. The ROESY correlations of H-10b/H-1 and H-4a/H-2 indicated the *β*-orientation of H-1 and *α*-orientation of H-2, which was further supported by its positive specific rotation ([*α*]*_D_*^23.3^ = +438.1) [22]. Therefore, compound **1** was identified as (+)-5,6-dehydrolycorine.

Compound **2** was obtained as a colorless oil. The HRESIMS displayed a pseudomolecular ion at *m/z* 410.1212 [*M*+Na]^+^ (calcd for C_20_H_21_NO_7_Na, 410.1216) consistent with a molecular formula of C_20_H_21_NO_7_, corresponding to 11 degrees of unsaturation. The IR absorption bands at 3385 and 1713 cm^−1^ are ascribable to the OH and the ester C=O groups, respectively. The ^1^H-NMR spectrum exhibited two singlets at *δ*_H_ 6.64 (s) and 6.83 (s) assigned to two *para*-position aromatic protons, a broad singlet at *δ*_H_ 5.88 ascribed to a methylenedioxy, two olefinic signals at *δ*_H_ 6.36 and 5.85 assigned to the H-1 and H-2, and two singlets at *δ*_H_ 2.11 (s) and 2.07 (s) ascribed to two acetoxyl Me groups. The ^13^C-NMR spectrum displayed 20 carbon resonances, including a phenyl (*δ*_C_ 102.9, 108.9, 124.7, 137.1, 146.4, 148.1), two Ac (*δ*_C_ 21.2 and 170.4; 20.2 and 170.1), a methylenedioxy (*δ*_C_ 101.2), three oxygenated methins (*δ*_C_ 67.3, 78.3 and 87.0), two olefinic carbons (*δ*_C_ 127.5 and 129.9), two CH_2_ (sp^3^), one CH (sp^3^) and a quaternary carbon (sp^3^) (Table 1). The above data resembled those of (+)-3*α*-methoxy-6*β*-acetyl- bulbispermine (**4**) [23] except for an acetoxyl group in **2** instead of the methoxyl group at C-3 in **4**, which was confirmed by HMBC correlations of H-3 (*δ*_H_ 3.91) with carbonyl group (*δ*_C_ 170.4) of acetoxyl group. The HMBC of the signal of proton at *δ*_H_ 4.51 (H-6) with *δ*_C_ 170.1 suggested that the other AcO group was located at C-6. The ROESY correlations of H-4a/H-3 and H-11/H-4a suggested both H-3 and H-11 to be *β*-orientation, and the ROESY correlation of H-6/H-12*α* indicated *α*-orientation for H-6 (Figure 2). Accordingly, the structure of **2** was established as (+)-3*α,*6*β*-diacetyl-bulbispermine.

Compound **3** corresponded to the molecular formula C_18_H_19_NO_6_, which was established by a quasimolecular ion peak in the HRESIMS. The general features of its IR and NMR spectra closely resembled those of **2**, except that the acetyl at C-3 in **2** were replaced by a hydroxyl in **3**, and an upfield shift of the signals for C-3 relative to that of **2** was observed. On the basis of the observation of NOESY data similar to those of **2**, the stereochemistry of **3** was expected to be the same. Accordingly, compound **3** was elucidated to be (+)-3*α*-hydroxy-6*β*-acetylbulbispermine.

Compound **5** was obtained as a colorless oil. Its positive HRESIMS spectrum showed a quasimolecular ion peak at *m/z* 316.1183 [*M*+H]^+^, consistent with the molecular formula C_17_H_17_NO, accounting for 10 degrees of unsaturation. The IR absorption bands at 1705 and 1655 cm^−1^ indicated the existence of ketones, while the UV absorption bands at 265 and 212 nm suggested a conjugated moiety. The ^l^H-NMR spectrum showed singlet signals for two aryl protons (*δ*_H_ 7.18 and 7.58), one olefinic proton (*δ*_H_ 5.80), one methylenedioxy (*δ*_H_ 6.07), and two vicinal methylenes [*δ*_H_ 2.70 (H-11) and 3.51, 3.70 (H-12)]. The ^13^C-NMR spectrum displayed one *N*-CH_3_, four CH_2_ and six CH groups (including three sp^3^ carbons and three sp^2^ carbons), and six sp^2^ quaternary carbons. The NMR data and the characteristic downfield signals of the carbon resonances for C-4a (*δ*_C_ 79.1), C-12 (*δ*_C_ 71.0), and *N*-CH_3_ (*δ*_C_ 56.2) indicated that **5** was a derivative of homolycorine-*α*-*N*-oxide [21,24]. The methylenedioxyl was positioned between C-8 and C-9, and the methyl at *N*-5, respectively, based on the HMBC correlations of the proton signal of methylenedioxy (*δ*_H_ 6.07) with C-8 (*δ*_C_ 151.8) and C-9 (*δ*_C_ 147.9), and of the methyl signal (δ_H_ 2.97) with C-4a and C-12, respectively (Figure 2). The ROESY correlation *N*-CH_3_/H-4a suggested *α*-orientation of the *N*-oxide. Thus, the structure of **5** was assigned the name (+)-8,9-methylenedioxyl-homolycorine-*N*-oxide.

Compound **7**, colorless needles, was assigned a molecular formula of C_15_H_13_NO_3_, based on the HRESIMS spectrum which showed a pseudomolecular ion at *m/z* 256.0977 [*M*+H]^+^ (calcd. 256.0974). Its ^13^C-NMR spectrum showed 15 carbon signals [NCH_3_ × 1, CH_2_ (sp^3^) × 2, CH (sp^2^) × 5 and C (sp^2^) × 7, Table 1]. The ^l^H-NMR spectrum showed singlet signals for two aryl protons (*δ*_H_ 7.02 and 6.69), one *N-*CH_3_ (*δ*_H_ 2.75), and an ABX system [*δ*_H_ 6.48 (1H, d, *J* = 3.2 Hz, H-1), 6.32 (1H, dd, *J* = 8.2, 3.2 Hz, H-3), and 6.63 (1H, d, *J* = 8.2 Hz, H-4)]. These spectral data showed similarities to those of 5,6-dihydro-5-methylphenanthridine [25]. The HMBC of C-2 (*δ*_C_ 154.1) with H-4 (*δ*_H_ 6.63) together the characteristic signals of the ABX system positioned the hydroxy group at C-2, which was further supported by the observation of downfield chemical shift of C-2 in **7**. Therefore, compound **7** was identified as 5,6-dihydro-5-methyl-2-hydroxyphenanthridine.

The cytotoxic activities of the isolated alkaloids were determined against eight human tumor cell lines, BEN-MEN-1 (meningioma), CCF-STTG1 (astrocytoma), CHG-5 (glioma), SHG-44 (glioma), U251 (glioma), HL-60 (human myeloid leukemia), SMMC-7721 (hepatocellular carcinoma), and W480 (colon cancer) using the modified MTT method. The *in vitro* cytotoxic activities of these compounds against human cell lines *a*re summarized in Table 2. Among the tested compounds, lycorine-type alkaloid **1** exhibited the most potent cytotoxic potential against all tested tumor cell lines, with IC_50_ values of 9.4–11.6 μM, except against BEN-MEN-1. Crinine-type alkaloids **2**–**4** showed significant cytotoxicities against HL-60 (IC_50_ < 10 μM), and moderate cytotoxicities against astrocytoma and glioma cell lines, CCF-STTG1, CHG-5, SHG-44 and U251 (10 μM < IC_50_ ≤ 30 μM). Homolycorine-type alkaloids **5**–**6** and **7** had no cytotoxic activities (IC_50_ > 80 μM).

Malaria is one of the most common vector-borne infectious diseases. This disease is caused by parasites of the genus *Plasmodium* and causes such symptoms as anemia, fever, chills, nausea, and in severe cases, coma and death. The effects of isolated alkaloids *in vitro* antimalarial activity were evaluated by using the drug-resistant D-6 strain and the drug-sensitive W-2 strain of *P. falciparum*. Lycorine-type alkaloid **1** possessed high antimalarial activities with low IC_50_ values (D-6: 2.3 μM; W-2 strain: 1.9 μM) (Table 3). Crinine-type alkaloids **2**–**4** showed moderate antimalarial activities, with values of 18.9, 17.9 and 21.3 μM for the D-6 strain and of 20.1, 19.3 and 23.4 μM for the W-2 strain, respectively. Homolycorine-type alkaloids **5**–**6** and **7** had no antimalarial activities.

**Table 1:** ^1^H-NMR data of compounds **1**–**3**and **5**in CDCl_3_ (*δ* in ppm and *J* in Hz).

No.	*δ* ^1^H (Hz)				*δ* ^13^C			
	1	2	3	5	1	2	3	5
1	4.66 (*dd*, 3.8, 3.2)	6.36 (*d*, 10.2)	6.38 (*d*, 10.2)	4.96 (*m*)	72.2	127.5	127.7	78.1
2	4.32 (*dd*, 5.2, 3.2)	5.85 (*dd*, 10.2, 5.0)	6.16 (*dd*, 10.2, 5.0)	2.53, 2.90 (*m*)	70.2	129.9	132.8	31.6
3	5.36 (*d*, 5.2)	3.91 (*m*)	4.27 (*m*)	5.80 (*dd*, 5.6, 5.0)	118.5	67.3	64.4	126.0
4	-	2.37 (*m*)	2.38 (*m*)	-	145.6	31.0	32.8	141.5
4a	4.46 (*d*, 13.6)	3.38 (*dd*, 13.8, 3.2)	3.40 (*dd*, 13.8, 3.2)	4.15 (*d*, 13.2)	60.4	58.5	57.8	79.1
6	8.84 (*s*)	4.51 (br *s*)	4.52 (br *s*)	-	163.2	87.0	87.1	166.8
6a	-	-	-	-	140.9	124.7	124.8	117.9
7	7.28 (*s*)	6.64 (*s*)	6.65 (*s*)	7.58 (*s*)	113.0	108.9	108.9	108.9
8	-	-	-	-	149.1	146.4	146.5	151.8
9	-	-	-	-	157.4	148.1	148.2	147.9
10	7.18 (*s*)	6.83 (*s*)	6.85 (*s*)	7.18 (*s*)	106.6	102.9	103.1	109.8
10a	-	-	-	-	128.3	137.1	137.2	136.3
10b	3.28 (*dd*, 13.6, 3.8)	-	-	3.63 (*dd*, 13.2, 2.8)	45.6	49.8	50.0	38.2
11	2.65, 2.88 (*m*)	3.96 (*m*)	3.97 (*m*)	2.70 (*m*)	34.4	78.3	78.4	26.5
12	4.23, 4.38 (*m*)	2.80, 3.32 (*m*)	2.81, 3.33 (*m*)	3.51, 3.70 (*m*)	59.0	58.8	58.9	71.0
OCH_2_O	6.17 (br *s*)	5.88 (br *s*)	5.90 (br *s*)	6.07 (br *s*)	104.7	101.2	101.2	102.1
*N-CH_3_*	-	-	-	2.97 (*s*)	-	-	-	56.2
OCH_3_	-	-	-	-	-	-	-	-
OCH_3_	-	-	-	-	-	-	-	-
*C*O_2_CH_3_	-	-	-	-	-	170.4	170.6	-
CO_2_*CH_3_*	-	2.11 (*s*)	2.12 (*s*)	-	-	21.2	21.6	-
*C*O_2_CH_3_	-	-	-	-	-	170.1	-	-
CO_2_*CH_3_*	-	2.07 (*s*)	-	-	-	20.2	-	-

**Table 2:** The cytotoxicity of compounds **1**–**7** against eight human tumor cell lines^a^.

	Cell lines							
	BEN-MEN-1	CCF-STTG1	CHG-5	SHG-44	U251	HL-60	SMMC-7721	W480
**1**		10.3 ± 0.9	10.2 ± 1.6	9.4 ± 1.3	11.8 ± 0.7	10.8 ± 1.6	10.5 ± 0.9	11.6 ± 1.1
**2**		27.1 ± 5.1	30.1 ± 4.4	27.1 ± 3.2	17.4 ± 2.1	7.3 ± 1.1	63.2 ± 11.8	51.1 ± 10.9
**3**		29.4 ± 4.1	29.4 ± 5.3	28.3 ± 2.7	15.8 ± 1.7	7.1 ± 0.9	66.8 ± 9.4	53.5 ± 12.4
**4**		29.7 ± 5.4	29.6 ± 6.3	29.1 ± 3.8	16.7 ± 2.6	8.6 ± 1.4	68.2 ± 12.3	50.1 ± 7.8
**5**	-	83.2 ± 13.7	-	-	-	-	86.2 ± 17.4	-
**6**	-	-	93.0 ± 21.1	-	-	-	85.0 ± 16.2	-
**7**	-	-	-	-	-	81.3 ± 15.7	-	-
Doxorubicin	17.8	24.7	21.8	33.7	28.4		37.6	14.1

^a^ Doxorubicin are expressed as IC_50_ values in nM, and compound **1**–**7** are expressed as IC_50_ values in μM. (-) IC_50_ > 100 μM.

**Table 3:** *In vitro* antimalarial activity against *Plasmodium falciparum* of compounds **1**–**7** ^a^.

	D-6	W-2
**1**	2.3	1.9
**2**	18.9	20.1
**3**	17.9	19.3
**4**	21.3	23.4
**5**	-	-
**6**	-	-
**7**	-	-
Chloroquine	9.8	6.7

^a^ Chloroquine data are expressed as IC_50_ values in nM, and compounds **1**–**7** are expressed as IC_50_ values in μM. (-) IC_50_ > 100 μM.

## 3. Experimental

### 3.1. General

Optical rotations were determined with a JASCO P2000 digital polarimeter (Tokyo, Japan). Ultraviolet (UV) and infrared (IR) spectra were obtained on JASCO V-650 and JASCO FT/IR-4100 spectrophotometers (Tokyo, Japan), respectively. The NMR spectra were measured in CDCl_3_ on a Bruker AM-600 spectrometer (Fällanden, Switzerland). Chemical shifts were reported using residual CDCl_3_ (*δ*_H_ 7.26 and *δ*_C_ 77.0 ppm) and CD_3_OD (*δ*_H_ 3.30 and *δ*_C_ 49.0 ppm) as internal standard. High resolution ESIMS spectra were obtained on a LTQ Orbitrap XL (Thermo Fisher Scientific, Waltham, MA, USA) spectrometer. Silica gel 60 (230–400 mesh, Merck, Darmstadt, Germany), LiChroprep RP-18 (Merck, 40–63 μm), and Sephadex LH-20 (Amersham Pharmacia Biotech, Roosendaal, The Netherlands) were used for column chromatography (CC). HPLC separation was performed on an instrument consisting of a Waters 600 controller, a Waters 600 pump, and a Waters 2487 dual λ absorbance detector, with a Prevail (250 × 10 mm i.d.) preparative column packed with C18 (5 μm). Precoated silica gel plates (Merck, Kieselgel 60 F254, 0.25 mm) and precoated RP-18 F_254s_ plates (Merck) were used for analytical thi*N-*layer chromatography analyses.

### 3.2. Plant Material

The bulbs of *L. radiata* were collected in April of 2011 in Lishui, a city of Zhejiang Province in China, and identified by one of the authors (Q.-J. Zhao). A specimen (201104001L) was deposited in the Herbarium of School of Pharmacy, Second Military Medical University, Shanghai, China.

### 3.3. Extraction and Isolation

The bulbs of *L. radiata* (10.5 kg) were cut into small pieces and were extracted with 80% ethanol (10 L) three times under reflux for 15 h and then concentrated under reduced pressure to give a crude extract (618.5 g). The crude extract was partitioned between equal volumes of chloroform and water to provide a chloroform-soluble fraction (110.6 g) and an aqueous layer. The chloroform-soluble fraction was further fractionated through a silica gel column (200–300 mesh) using increasing volumes of acetone in petroleum ether (100:1, 50:1, 30:1, 15:1, 10:1, 7:1, 5:1, 3:1, 1:1, V/V) as eluents to give 12 fractions according to TLC analysis. Fraction 5 (5.3 g) was applied to an ODS MPLC column (100 g) and eluted with MeOH-H_2_O (20:80, 30:70, 40:60, each 500 mL) to yield four subfractions (Fr. 5-1 and Fr. 5-4). Subfraction 5-2 (358 mg) was purified by a preparative RP-HPLC (ODS column, 250 × 20 mm) using MeOH/H_2_O (26:74) as mobile phase to obtain **2** (73 mg) and **7** (64 mg). Subfraction 5-3 (515 mg) was chromatographed by a Sephadex LH-20 column eluted with MeOH/H_2_O (50:50), and purifed by a preparative RP-HPLC (ODS column, 250 × 20 mm) using MeOH/H_2_O (30:70) as mobile phase to yield **4** (73 mg) and **5** (68 mg). Fraction 6 (3.3 g) was applied to an ODS column eluted with MeOH/H_2_O (30:70, 40:60, 50:50) to provide 4 Subfraction (Fr. 6-1 and Fr. 6-4). Subfraction 6-2 (119 mg) was purified by a preparative RP-HPLC (ODS column, 250 × 20 mm) eluted with MeOH/H_2_O (22:78) to get **6** (57 mg). Subfraction 6-3 (MeOH-H_2_O 20:80, 303 mg) was repeatedly chromatographed on silica gel (chloroform:methanol, 20:1 → 10:1) and then purifed by a Sephadex LH-20 column eluted with MeOH/H_2_O (50:50) to afford **1** (68 mg). Subfraction Subfraction 6-4 was purified by a preparative RP-HPLC (ODS column, 250 × 20 mm) eluted with MeOH/H_2_O (23:77) to get **3** (73 mg).

(*+*)*-5,6-Dehydrolycorine* (**1**): Yellow amorphous powder. [*α*]*_D_*^23.3^ = +438.1 (*c* = 0.11, MeOH). UV (CDCl_3_) λ_max_(log *ε*): 374 (4.05), 309 (3.83), 253 (4.22), 212 (4.65) nm. IR (KBr) *ν*_max_ 3410, 3355, 1645, 1605, 1590, 1502, 1275, 1035, 923 cm^−1^. ^1^H-NMR and ^13^C-NMR data, see Table 1. ESI-MS *m/z*: 286 ([*M*]^+^). HR-ESI-MS (pos.) *m/z*: 286.1075 ([*M*]^+^, C_16_H_16_NO_4_^+^. calc. 286.1074).

(+)-*3α,6β-Diacetylbulbispermine* (**2**): Colorless oil. [*α*]*_D_*^23.3^ = +33.7 (*c* = 0.16, MeOH). UV (CDCl_3_) λ_max_(log *ε*): 292 (3.73), 240 (3.90) nm. IR (KBr) *ν*_max_ 3385, 2902, 1713, 1483, 1250, 1060, 933, 870 cm^−1^. ^1^H-NMR and ^13^C-NMR data, see Table 1. EI-MS *m/z*: 387 ([*M*]^+^). HR-ESI-MS (pos.) *m/z*: calc. 410.1212 ([*M* + Na]^+^, C_20_H_21_NO_7_Na. calc. 410.1216).

(+)-*3α-Hydroxy-6β-acetylbulbispermine* (**3**): Colorless oil. [*α*]*_D_*^23.3^ = +23.6 (*c* = 0.10, MeOH). UV (CDCl_3_) λ_max_(log *ε*): 291 (3.78), 240 (3.87) nm. IR (KBr) *ν*_max_ 3386, 2898, 1712, 1485, 1248, 1055, 930 cm^−1^. ^1^H-NMR and ^13^C-NMR data see Table 1. EI-MS *m/z*: 345 ([*M*]^+^). HR-ESI-MS (pos.) *m/z*: calc. 368.1112 ([*M* + Na]^+^, C_18_H_19_NO_6_Na. calc. 368.1110).

(+)-*8,9-Methylenedioxyl-homolycorine-N-oxide* (**5**): Colorless oil. [*α*]*_D_*^23.3^ = +143.9 (*c* = 0.14, MeOH). UV (CDCl_3_) λ_max_(log *ε*): 380 (2.72), 308 (3.55), 265 (3.78), 212 (4.56) nm. IR (KBr) *ν*_max_2945, 1705, 1655, 1600, 1453, 1311, 1224, 1064, 911 cm^−1^. ^1^H-NMR and ^13^C-NMR data, see Table 1. EI-MS *m/z*: 315 ([*M*]^+^). HR-ESI-MS (pos.) *m/z*: calc. 316.1183 ([*M* + H]^+^, C_17_H_18_NO_5_. calc. 316.1185).

*5,6-Dihydro-5-methyl-2-hydroxyphenanthridine* (**7**): Colorless needles. [*α*]*_D_*^23.3^ = +43.1 (*c* = 0.05, MeOH). UV (CDCl_3_) λ_max_(log *ε*): 380 (2.65), 308 (3.55), 263 (3.70), 210 (4.40) nm. IR (KBr) *ν*_max_ 3410, 1710, 1640, 1602, 1460, 1255, 1035, 915 cm^−1^. ^1^H-NMR *δ*_H_: 6.48 (1H, d, *J* = 3.2 Hz, H-1), 6.32 (1H, dd, *J* = 8.2, 3.2 Hz, H-3), 6.63 (1H, d, *J* = 8.2 Hz, H-4), 4.21 (2H, br s, H-6), 7.02 (1H, s, H-7), 6.69 (1H, s, H-10), 2.75 (3H, s, *N*-Me), 5.99 (2H, s, OCH_2_O); ^13^C-NMR *δ*_C_: 113.6 (C-1), 154.1 (C-2), 117.2 (C-3), 113.0 (C-4), 138.9 (C-4a), 63.8 (C-6), 133.7 (C-6a), 109.9 (C-7), 147.7 (C-8), 147.6 (C-9), 107.3 (C-7), 110.4 (C-10), 131.0 (C-10a), 117.5 (C-10b), 31.1 (*N*-Me). EI-MS *m/z*: 255 ([*M*]^+^). HR-ESI-MS (pos.) *m/z*: 256.0977 ([*M* + H]^+^, C_15_H_14_NO_3_. calc. 256.0974).

### 3.4. Cytotoxicity Assay *in Vitro*

The cytotoxic activities of the isolated compounds were determined using the revised MTT method [26,27] against BEN-MEN-1 (meningioma), CCF-STTG1 (astrocytoma), CHG-5 (glioma), SHG-44 (glioma), U251 (glioma), HL-60 (human myeloid leukemia), SMMC-7721 (hepatocellular carcinoma), and W480 (colon cancer). Doxorubicin was used as the positive control. Cancer cells (4 × 10^3^ cells) suspended in 100 μL/well of DMEM medium containing 10% fetal calf serum were seeded onto a 96-well culture plate. After 24 h pre-incubation at 37 °C in a humidified atmosphere of 5% CO_2_/95% air to allow cellular attachment, various concentrations of test solution were added and cells were incubated for 48 h under the above conditions. At the end of the incubation, 10 μL of tetrazolium reagent was added into each well followed by further incubation at 37 °C for 4 h. The supernatant was decanted, and DMSO (100 μL/well) was added to allow formosan solubilization. The concentrations of the assayed compounds were 0.04, 0.2, 1.0, 5, 25, and 125 μM, respectively. The optical density (OD) of each well was detected using a microplate reader at 550 nm and for correction at 595 nm. Each determination represented the average mean of six replicates. The 50% inhibition concentration (IC_50_ value) was determined by non-linear regression with Graphpad Prism software version 4.0 (GraphPad Software, Inc., San Diego, CA, USA) and was used as criteria to judge the cytotoxicity. All the IC_50_ results represent an average of a minimum of three experiments and were expressed as means ± standard deviation (SD). All cell lines were purchased from the Cell Bank of the Shanghai Institute of Biochemistry & Cell Biology, Chinese Academy of Sciences (Shanghai, China). Other reagents were purchased from Shanghai Sangon Biological Engineering Technology & Services Co., Ltd (Shanghai, China). 

### 3.5. Assay for *in Vitro* Antimalarial Activity against Plasmodium Falciparum

*P. falciparum* strains D-6 (drug-resistant) and W-2 (drug-sensitive) were cultured in human erythrocytes in RPMI medium (RPMI-1640 with 25 mM HEPES buffer, 24 mM NaHCO_3_, 0.2% glucose, 0.05% L-glutamine, 50 μg/mL hypoxanthine, and 25μg /mL gentamicin) supplemented with 10% human plasma at 37 °C, under 93% N_2_, 4% CO_2_, and 3% O_2_ [4,10]. Antimalarial activity of the test compound was determined from the dose–response curve using the parasite lactate dehydrogenase (pLDH) assay according to the method of Makler [4,10]. The concentrations of the assayed compounds were 0.02, 0.1, 0.5, 2.5, 12.5, and 62.5 μM, respectively. One hundred and ninety microliters of asynchronous parasites (2.0% hematocrit and 0.5 or 1% parasitemia) was seeded into a 96-well microplate and 10 μL of test compound solution (dissolved in 25% ethanol or 5% DMSO) was added. After incubating at 37 °C for 72 h under 93% N_2_, 4% CO_2_, and 3% O_2_, the microplate was immediately frozen at −20 °C for 18 h. Then, the microplate was thawed at 37 °C and 20 μL of the hemolyzed parasite suspension was transferred to another microplate containing 100 μL of Malstat reagent. The plate was further incubated for 15 min at room temperature, and 20 μL of a 1:1 mixture of nitroblue tetrazolium and phenazine ethosulfate (2 mg and 0.1 mg/mL, respectively) was added to each well. After incubation for 2 h at room temperature in the dark, the blue formazan product was measured at 655 nm with iEMS microplate reader MF (Labsystems, Helsinki, Finland). The 50% inhibitory concentration (IC_50_) value was determined by non-linear regression with Graphpad Prism software version 4.0 (GraphPad Software, Inc.). It was used as criteria to judge the activity (active: IC_50_ ≤ 10 μM; moderately active: 10 μM < IC_50_ ≤ 30 μM; not active: IC_50_ > 30 μM). *P. falciparum* strains were purchased from the Cell Bank of the Shanghai Institute of Biochemistry & Cell Biology, Chinese Academy of Sciences.

## 4. Conclusions

Five new Amaryllidaceae alkaloids: (+)-5,6-dehydrolycorine (**1**), (+)-3*α,6β*-diacetylbulbispermine (**2**), (+)-3*α*-hydroxy-6*β*-acetylbulbispermine (**3**), (+)-8,9-methylenedioxyl-homolycorine-*N*-oxide (**5**), and 5,6-dihydro-5-methyl-2-hydroxyphenanthridine (**7**), and two known compounds, (+)-3*α*-methoxy-6*β*-acetylbulbispermine (**4**) and (+)-homolycorine- *N*-oxide (**6**), were isolated from the 80% ethanol extract of the bulbs of *Lycoris radiata*. All the alkaloids were evaluated *in vitro* for cytotoxic properties against BEN-MEN-1 (meningioma), CCF-STTG1 (astrocytoma), CHG-5 (glioma), SHG-44 (glioma), U251 (glioma), HL-60 (human myeloid leukemia), SMMC-7721 (hepatocellular carcinoma), and W480 (colon cancer) and antimalarial activity against two strains of *P. falciparum* (D-6: drug-resistant and W-2: drug-sensitive). Alkaloids **1**–**4** showed significant cytotoxic activities against HL-60 (IC_50_ values < 10 μM). Lycorine-type alkaloid **1** exhibited the most potent cytotoxicities and crinine-type alkaloids **2**–**4** possessed moderate cytotoxicities against astrocytoma and glioma cell lines, CCF-STTG1, CHG-5, SHG-44 and U251 (10 μM < IC_50_ value ≤ 30 μM). Homolycorine-type alkaloids **5**–**6** and **7** had no cytotoxic activities (IC_50_ values > 80 μM). In addition, lycorine-type alkaloid **1** has antimalarial activities against the two strains of *P. falciparum.*
